# The expression of p53-induced protein with death domain (Pidd) and apoptosis in oral squamous cell carcinoma

**DOI:** 10.1038/sj.bjc.6603745

**Published:** 2007-04-17

**Authors:** G Bradley, S Tremblay, J Irish, C MacMillan, G Baker, P Gullane, S Benchimol

**Affiliations:** 1 Faculty of Dentistry, University of Toronto, Toronto, ON, Canada; 2 The Ontario Cancer Institute, University of Health Network, Toronto, ON, Canada; 3 Department of Otolaryngology/Head and Neck Surgery, University Health Network, Toronto, ON, Canada; 4 Department of Pathology, University Health Network, Toronto, ON, Canada; 5 Department of Dentistry, Mount Sinai Hospital, Toronto, ON, Canada; 6 Department of Medical Biophysics, University of Toronto, Toronto, ON, Canada; 7 Department of Biology, York University, Toronto, ON, Canada

**Keywords:** Pidd, apoptosis, p53, oral, carcinoma

## Abstract

The *Pidd* (p53-induced protein with death domain) gene was shown to be induced by the tumour suppressor p53 and to mediate p53-dependent apoptosis in mouse and human cells, through interactions with components of both the mitochondrial and the death receptor signalling pathways. To study the role of *Pidd* in clinical tumours, we measured its expression by quantitative reverse transcription-PCR in microdissected oral squamous cell carcinomas (OSCC) with and without *p53* mutation. Tumour cell apoptosis was assessed by *in situ* terminal deoxynucleotidyl transferase-mediated dUTP nick-end labelling. Tumour proliferation was assessed by immunohistochemical staining for the Ki-67 antigen. We found a wide range of *Pidd* expression among OSCC. Statistical analysis revealed an association between *Pidd* expression and apoptotic index (Mann–Whitney test, *P*<0.001), consistent with a role of *Pidd* in apoptosis in this tumour type. Furthermore, we showed a positive correlation between apoptotic index and proliferative index that has not been previously described for OSCC. There was no correlation between *Pidd* expression and the *p53* mutation status of these tumours, suggesting that *Pidd* expression may be regulated by p53-independent mechanisms. Further characterisation of these molecular defects in the control of proliferation and apoptosis should help in developing treatments that target OSCC according to their biological properties.

*Pidd* (p53-induced protein with death domain) was characterised as a p53-induced gene that encodes a death domain containing protein, and has been shown to mediate p53-dependent apoptosis in a variety of cell types (Lin *et al*, 2000). The structural features of the Pidd protein suggest that it may function as an adaptor protein that links other apoptotic signalling molecules. Indeed, the Pidd protein has been shown to form a complex with signalling molecules in both the death receptor and mitochondrial pathways (Telliez *et al*, 2000; Tinel and Tschopp, 2004; Berube *et al*, 2005). Currently, little is known about the pathophysiologic role of Pidd in clinical cancers, and whether Pidd expression may be used as a marker of apoptotic function that can guide cancer therapy.

Squamous cell carcinoma is the predominant malignant tumour in the oral cavity. The development of oral squamous cell carcinoma (OSCC) is strongly associated with smoking and excessive alcohol consumption, although a minority of OSCC develops in non-smokers and non-drinkers (Koch and McQuone, 1997). The histopathological appearance of OSCC varies from well-differentiated tumours with discrete islands of keratinising carcinoma to poorly differentiated, non-keratinising tumours that show diffuse invasion by single malignant cells or small groups of cells (Anneroth *et al*, 1987). Several molecular abnormalities have been described for OSCC, particularly mutation of the *p53* tumour suppressor gene (Forastiere *et al*, 2001; Le and Giaccia, 2003), but the molecular biology of OSCC of different degrees of differentiation is not well understood.

Abnormalities in the regulation of cell proliferation and apoptosis play an important role in cancer development (Hanahan and Weinberg, 2000). In previous studies that examined apoptosis and their markers in OSCC, the expression of anti-apoptotic proteins such as Bcl-2 was reported to be strongest in poorly differentiated carcinoma, whereas pro-apoptotic proteins such as Bax and high apoptotic index (AI) correlate with well-differentiated carcinoma (Jordan *et al*, 1996; Xie *et al*, 1999; Stoll *et al*, 2000). This would suggest that apoptosis has not been effectively evaded in well-differentiated, keratinising squamous cell carcinomas with low proliferative index (PI) and makes it difficult to account for the growth of these tumours. Further study of additional apoptotic markers and pathways are needed to investigate the relative importance of proliferation and apoptosis in OSCC. The present study was undertaken to examine the relationship between expressions of the apoptotic protein Pidd, tumour cell apoptosis and proliferation.

## MATERIALS AND METHODS

### Patients and tumour samples

Fifty-five samples of squamous cell carcinoma of the oral cavity were collected at the time of tumour resection at the University Health Network, between 1996 and 2000. The protocol for tissue collection and analysis has been approved by the University Health Network Research Ethics Board. There were 35 male patients and 20 female patients, aged 25–82, with a median age of 60. There were five patients under the age of 40. Thirty patients were smokers, nine were former smokers and 15 had never smoked; the smoking history of one patient was not available. Thirty-seven tumours were from the lateral or ventral tongue, 12 from the floor of mouth, four from buccal mucosa and two were from gingiva/alveolar mucosa. All samples were previously untreated OSCC. Tumours were classified at diagnosis according to the TNM classification (Sobin and Wittekind, 2002) as stage II (17 cases), stage III (15 cases) and stage IV (23 cases).

Immediately after tumour excision, unfixed tumour samples were embedded in OCT compound (Tissuetek, Immucor, ON, Canada), frozen in isopentane prechilled in liquid nitrogen and stored at −80°C until used.

Twelve samples of normal oral mucosa from the buccal mucosa and gingiva were obtained as surgical waste from patients undergoing oral surgery. The patients ranged in age from 16 to 39. Unfixed mucosal samples were frozen in OCT compound following the same procedure as for carcinoma samples. All 12 samples were histologically verified to be free of epithelial dysplasia and carcinoma, and were used to set a threshold to separate low *Pidd* expression from high *Pidd* expression in the carcinomas for this study.

### Laser capture microdissection and RNA isolation

To assess *Pidd* expression in OSCC and normal oral epithelium without the confounding effect of its expression in inflammatory and stromal cells that are also present within the samples, gene expression analysis was carried out with RNA prepared from microdissected cryostat sections. Carcinoma cells and normal epithelial cells, respectively, were collected from cryostat sections by laser capture microdissection (PixCell II, Arcturus, Mountain View, CA, USA), following protocols provided by the manufacturer. Approximately 5000 cells were harvested from two to four sections for each sample. RNA was isolated using a procedure specially adapted for laser capture microdissection samples (Stratagene Absolutely RNA Microprep kit, La Jolla, CA, USA). Following removal of DNA by in-column DNase digestion, the RNA was eluted in 30 *μ*l of elution buffer and stored at −80°C until use.

### Quantitative reverse transcription-polymerase chain reaction analysis of *Pidd* expression

A 172-bp fragment at the 3′ end of the human *Pidd* mRNA sequence was amplified in a quantitative real-time, one-step reverse transcription-polymerase chain reaction (RT-PCR) using the ABI PRISM 7700 sequence detection system (Perkin-Elmer Applied Biosystems, Foster City, CA, USA). A 157-bp fragment of 18S RNA was amplified in a parallel reaction using the same RNA template, to normalise *Pidd* gene expression values for differences in the input of RNA template between samples (Wang *et al*, 2002). The single-step RT-PCR reaction mix consisted of QuantiTect SYBR Green RT-PCR 2 × Master Mix, 5 × Q-solution, QuantiTect RT Mix (QuantiTect SYBR Green RT-PCR kit, Qiagen Inc., Mississauga, ON, Canada), forward and reverse primers at a final concentration of 0.5 *μ*M each, template RNA and nuclease-free water for a total reaction volume of 50 *μ*l. The primers for RT-PCR of *Pidd* were: forward 5′ CTGGATGAGCAGATCCGTCAC 3′, reverse 5′ GGATGCTGTCCTGGTACTTGC 3′. Primers for 18S RNA were: forward 5′ GCCTGGATACCGCAGCTAG 3′, reverse 5′ TTCGCTCTGGTCCGTCTTG 3′. The reaction protocol was as follows: 30 min at 50°C for reverse transcription, 15 min at 95°C to inactivate the reverse transcriptase, activate the HotStarTaq DNA polymerase and denature the DNA, and 40 cycles of 15 s at 94°C, 30 s at 58°C and 60 s at 72°C. Melting curve analysis was performed after each RT-PCR run to ensure that the fluorescence measurements were based on a single amplified product (ABI PRISM 7700 Sequence Detection System protocol).

*Pidd* expression was determined relative to the oral carcinoma cell line UTSCC24A (kindly provided by R Grenman, University of Turku, Finland), the expression in this cell line being set at 1. In each run of real time RT-PCR, a dilution series of UTSCC24A RNA was included with the carcinoma and mucosal RNA samples and a standard curve of log template *vs* C_T_ (threshold cycle of amplification) was generated. The amount of *Pidd* RNA in the carcinoma and mucosal RNA samples was obtained by interpolation of the standard curve, following the established protocol (ABI PRISM 7700 Sequence Detection System protocol). Each RNA sample was measured in duplicate and the average was calculated.

### TUNEL assay for apoptosis in tissue sections

Apoptotic cells in cryostat sections of OSCC and normal oral mucosa were identified by *in situ*-end labelling of DNA strand breaks (*In situ* Cell Death Detection Kit, Roche Applied Science, Penzberg, Germany), according to the manufacturer's instructions. Briefly, cryostat sections were fixed in 4% formaldehyde in PBS (pH 7.4) for 20 min at 15–25°C, washed in PBS, incubated with 3% H_2_O_2_ in methanol for 10 min at 15–25°C to block endogenous peroxidase activity, rinsed in PBS and permeabilised for 2 min with 0.1% Triton X-100 and 0.1% sodium citrate at 4°C. The sections were rinsed and then incubated with terminal deoxynucleotidyl transferase (TdT) in a buffer that contained fluorescein-tagged dUTP, for 1 h at 37°C in a humidified chamber. The sections were rinsed and next incubated with antifluorescein antibody conjugated with horseradish peroxidase, for 30 min at 37°C in a humidified chamber, followed by reaction with the Nova Red substrate mixture with hydrogen peroxide (Vector Laboratories, Burlington, ON, Canada). The sections were counterstained with haematoxylin. Negative controls consisted of sections incubated with enzyme dilution buffer instead of the TdT enzyme.

Apoptotic cells are identified by dark brownish-red staining from the terminal deoxynucleotidyl transferase-mediated dUTP nick-end labelling (TUNEL) reaction over the whole nucleus or multiple globular bodies in place of the nucleus, together with the appearance of cell shrinkage. They are typically seen singly and surrounded by non-apoptotic cells, and can be distinguished from necrotic cells, which show less intense brown staining, cell swelling and rupture and form a confluent area within the tissue often with infiltration of inflammatory cells (Darzynkiewicz *et al*, 1997; Garrity *et al*, 2003; Kroemer *et al*, 2005).

### Immunohistochemical staining for proliferating cells

Staining with the MIB-1 antibody against the Ki-67 antigen was used to identify cells that were in the G1 through M phases of the cell cycle (Cattoretti *et al*, 1992). Immunohistochemical staining was performed according to previously established procedures (Bradley *et al*, 2001). Briefly, cryostat sections were placed on silanised slides and fixed with 3.7% buffered formaldehyde (10 min at room temperature), rinsed in PBS, treated with absolute methanol (4 min at −20°C) and then acetone (2 min at −20°C) and rinsed again with PBS. Endogenous peroxidase activity was blocked with 0.3% H_2_O_2_ for 10 min and nonspecific binding was blocked by incubation with 10% normal horse serum for 30 min. Sections were incubated with the mouse monoclonal antibody MIB-1 (Dako Cytomation, Copenhagen; antibody was used at 1–2 *μ*g ml^−1^ in 0.1% BSA/PBS) for 1 h, washed with PBS, incubated with biotinylated horse anti-mouse IgG (Vector Laboratories, Burlington, ON, Canada) for 30 min, washed again, and incubated with avidin-biotin horseradish peroxidase complex (Vector Laboratories) for 30 min. The peroxidase label was visualised with the Nova Red substrate kit (Vector Laboratories). All incubations were carried out at room temperature. Sections were counterstained with haematoxylin. Negative controls were run by omitting the primary antibody.

### Image analysis

The amount of TUNEL staining and MIB-1 staining in the sections was quantitated with the aid of an image analysis program (Image ProPlus, version 4.1, Media Cybernetics, MD, USA). For TUNEL staining, approximately 5000 tumour cells were examined and the number of apoptotic cells, identified by the criteria given above, was counted to give the AI, which is the percent of apoptotic cells. For MIB-1 staining, at least 2000 tumour cells were examined and the number of MIB-1 positive nuclei was counted to give the PI, which is the percent of MIB-1 positive cells.

### Sequencing of the p53 gene

DNA extraction from tumour samples, amplification of the *p53* gene by PCR and direct sequencing of the amplified DNA were performed according to previously established procedures (Bradley *et al*, 2001). Briefly, DNA was extracted from cryostat sections of OSCC after non-malignant tissues have been removed under a dissecting microscope. The coding section of the *p53* gene was amplified from genomic DNA in three segments that encompassed exons 2–4, 5–9, and 10 and 11, respectively. DNA sequencing of the amplified *p53* fragments was performed with an automated sequencer (ABI Prism 377 DNA Sequencer, Applied Biosystems, Foster City, CA, USA). All mutations were verified with a second PCR amplification and repeat sequencing.

### Statistical analysis

The data were analysed with SPSS v.15 (Statistical Package for Social Sciences). Differences between groups were examined using the Mann–Whitney rank-sum test or the Kruskal–Wallis test. A two-tailed test was used in each analysis. *P*<0.05 was considered to be statistically significant.

## RESULTS

### *Pidd* expression is associated with apoptosis in OSCC

Our study of OSCC showed a wide range of *Pidd* expression levels, from 0.36 to 4.69 relative units, compared with *Pidd* expression in normal oral epithelium from individuals with no history of carcinoma, which varied from 0.56 to 1.20 relative units. To investigate the functional significance of the variation in *Pidd* expression among cases of OSCC, we measured the amount of apoptosis by *in situ* TUNEL assay on tumour sections. Terminal deoxynucleotidyl transferase-mediated dUTP nick-end labelling was performed on serial sections of the same tumour blocks that were used for laser capture microdissection, RNA preparation and real-time RT-PCR, to allow a valid comparison between AI and *Pidd* expression. Measurement of *Pidd* expression and *in situ* TUNEL analysis was performed on 43 cases of OSCC.

**Figure 1:**
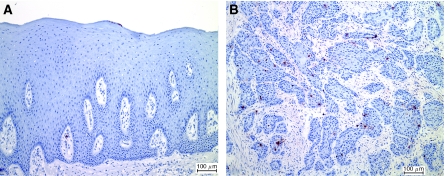
Terminal deoxynucleotidyl transferase-mediated dUTP nick-end labelling staining of normal oral mucosa (**A**) and oral squamous carcinoma (**B**). Apoptotic cells are stained brownish-red; sections are counterstained with haematoxylin. Bar indicates 100 *μ*m.

Terminal deoxynucleotidyl transferase-mediated dUTP nick-end labelling staining of normal oral mucosa revealed rare apoptotic cells within the epithelium (Figure 1A) and also within the connective tissue, the latter appearing to be apoptotic lymphocytes. The amount of apoptosis seen in carcinomas varied (Figure 1B), with a range of apoptotic indices from 0.03 to 2.25%. The amount of apoptosis that we observed in the TUNEL assay was generally similar to that reported by others (Xie *et al*, 1999; Macluskey *et al*, 2000; Stoll *et al*, 2000). The late stages of apoptosis that result in the distinctive morphology of an apoptotic cell are thought to occur rapidly, followed by phagocytic removal of the apoptotic bodies by neighbouring cells. Thus, an AI around 1% is interpreted as a significant amount of cell death (Darzynkiewicz *et al*, 1997; Lippens *et al*, 2005).

**Figure 2:**
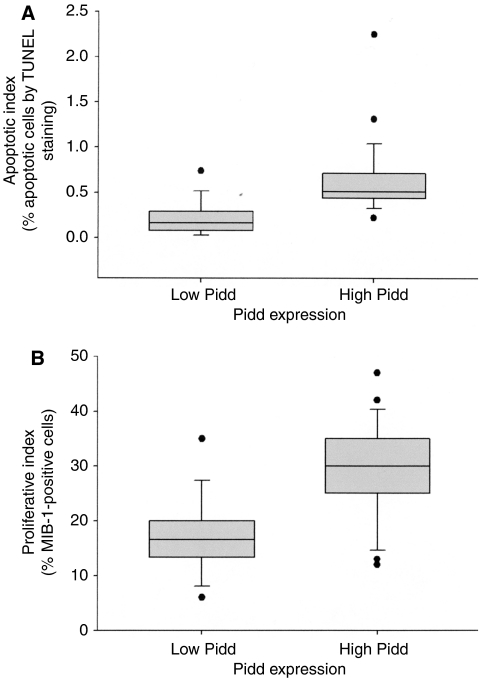
(**A**) Distribution of apoptotic indices in OSCC with low *Pidd* expression (*N*=16) and OSCC with high *Pidd* expression (*N*=27). Apoptotic index is measured as the percentage positive cells in an *in situ* TUNEL assay. Low *Pidd* expression is defined as relative *Pidd* expression of 1.2 or less, high *Pidd* expression is defined as relative *Pidd* expression of more than 1.2 (see Results and Discussion). Each vertical box shows the median and interquartile range, the bars show the 10th and 90th percentiles and the dots indicate apoptotic indices outside the 10th and 90th percentiles. There is a significant difference in AI between OSCC with low *Pidd* expression and those with high *Pidd* expression (Mann–Whitney rank-sum test, *P*<0.001). (**B**) Distribution of proliferative indices in OSCC with low *Pidd* expression (*N*=16) and OSCC with high *Pidd* expression (*N*=27). Proliferative index is measured as the percentage of cells that are MIB-1 positive by immunohistochemical staining. Low *Pidd* expression is defined as relative *Pidd* expression of 1.2 or less, high *Pidd* expression is defined as relative *Pidd* expression of more than 1.2. Each vertical box shows the median and interquartile range, the bars show the 10th and 90th percentiles and the dots indicate proliferative indices outside the 10th and 90th percentiles. There is a significant difference in PI between OSCC with low *Pidd* expression and those with high *Pidd* expression (Mann–Whitney rank-sum test, *P*<0.001).

Figure 2A compares the apoptotic indices of OSCC with low and high *Pidd* expression. Low *Pidd* expression was defined as expression at or below that found in normal mucosa, which was 1.2 relative units or less. Oral squamous cell carcinomas with low *Pidd* expression (16 out of the 43 cases) have significantly lower apoptotic indices compared to OSCC with high *Pidd* expression (*P*<0.001).

### *Pidd* expression is associated with proliferation in OSCC

**Figure 3:**
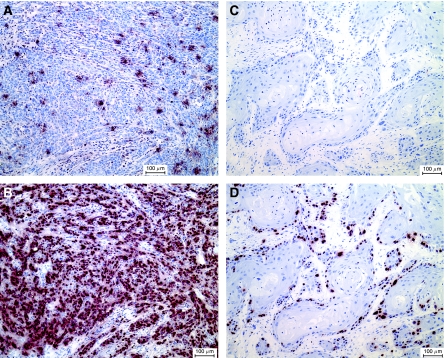
(**A** and **B**) *In situ* TUNEL for apoptotic cells (**A**) and MIB-1 staining for proliferating cells (**B**) in an OSCC with Pidd overexpression compared to normal oral epithelium (Pidd expression=4.09 relative units, AI=1.31%, PI=42%). (**C** and **D**) *In situ* TUNEL for apoptotic cells (**C**) and MIB-1 staining for proliferating cells (**D**) in an OSCC with Pidd underexpression compared to normal oral epithelium (Pidd expression=0.36 relative units, AI=0.03%, PI=19%). Apoptotic cells (**A** and **C**) and MIB-1 expressing cells (**B** and **D**) are stained brownish-red and the sections are counterstained with haematoxylin. Bar indicates 100 *μ*m.

We noted that the OSCC samples with high *Pidd* expression and high AI were typically non-keratinising carcinomas, whereas OSCC at the opposite end of the spectrum of *Pidd* expression showed large islands with squamous differentiation and central keratinisation. Since the non-keratinising histological appearance is usually associated with a high PI as measured by MIB-1 staining, we compared the proliferative indices of OSCC with low (1.2 relative units or less) and high (more than 1.2 relative units) *Pidd* expressions (Figure 2B). Oral squamous cell carcinomas with low *Pidd* expression have significantly lower proliferative indices compared to those with high *Pidd* expression (*P*<0.001). Figure 3A and B illustrates the association of high AI, high PI and non-keratinising histological appearance in an OSCC with high *Pidd* expression, in contrast with Figure 3C and D, which shows low AI and low PI in a well-differentiated, keratinising OSCC with low *Pidd* expression.

### Correlation of *Pidd* expression with clinical data

We compared *Pidd* expression and AI in OSCC of smokers or former smokers, with OSCC in patients who have never smoked. Of the 43 OSCC for which we measured *Pidd* expression and AI, 30 were from smokers or former smokers and 12 were from non-smokers. The smoking history of one patient was not available. Previous studies have identified differences in molecular pathology between OSCC in smokers and those in non-smokers. *p53* mutation is common in OSCC of smokers, but less often detected in carcinomas of those who never smoked. Loss of heterozygosity at selected microsatellite loci is more frequent in OSCC of smokers than in non-smokers (Koch *et al*, 1999). However, our data show no significant correlation between smoking history and either *Pidd* expression or AI. For smokers or former smokers, the median *Pidd* expression was 1.55 and median AI was 0.44, whereas for non-smokers median *Pidd* expression was 1.24 and median AI was 0.38.

We sought to determine if *Pidd* expression, AI and PI were related to disease stage at presentation. Of 43 cases of OSCC, 16 patients had stage IV disease, 12 had stage III, 15 had stage II and none had stage I disease. There was no correlation between stage of disease and *Pidd* expression, AI or PI (Table 1). In particular, there were keratinising OSCC with low *Pidd* expression, low PI and low AI that presented as extensive disease at the primary site and multiple metastases to regional lymph nodes.

**Figure 4:**
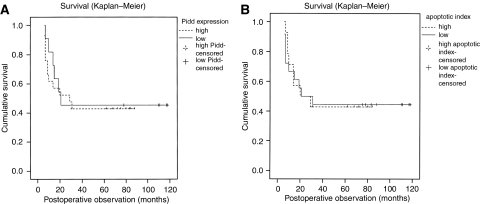
(**A** and **B**) Kaplan–Meier plot for disease-free survival by *Pidd* expression (**A**) and by AI (**B**) for 32 cases of OSCC. Low *Pidd* expression is defined as relative expression of 1.2 or less, high *Pidd* expression is defined as relative expression of more than 1.2. Low AI is defined as AI that is at or below the median AI for this group of OSCC (median AI is 0.45), and high AI is defined as AI that is above the median value. There is no significant difference in the pattern of disease-free survival by *Pidd* expression or by AI (log-rank test).

Clinical follow-up data were obtained for 36 out of the 43 patients for whom *Pidd* expression, AI and PI of the carcinoma were measured. Eighteen patients died of locoregional disease recurrence and/or distant metastases, after a mean follow-up period of 13 months (range of 6–31 months). One patient was found to have recurrent disease at 29 months after surgery and died of a cerebrovascular accident 5 months later. Fourteen patients had no evidence of disease after a mean follow-up period of 84 months (range of 31–118 months). Three patients were lost to follow-up within 24 months after surgery. For the 32 patients who were observed for an adequate period (more than 2 years) after surgical resection, the pattern of disease-free survival was compared between cases with high Pidd expression and those with low Pidd expression, and also between cases with high AI and low AI. There was no statistically significant difference in survival by Pidd expression or AI (Figure 4A and B).

### *Pidd* expression does not correlate with *p53* mutational status

**Figure 5:**
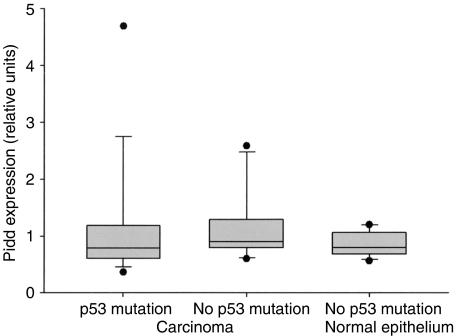
Distribution of *Pidd* expression in OSCC with *p53* mutation (*N*=16), OSCC with no detected *p53* mutation (*N*=8) and normal oral mucosa (*N*=12). Each vertical box shows the median and interquartile range, the bars show the 10th and 90th percentiles and the dots indicate *Pidd* expression values outside the 10th and 90th percentiles. There is no significant difference in *Pidd* expression between OSCC with p53 mutation and OSCC with no p53 mutation (Mann–Whitney rank-sum test, *P*=0.426).

*Pidd* mRNA levels were measured by quantitative real-time RT-PCR in 24 cases of OSCC with known *p53* mutation status. There were 16 cases with *p53* mutation, including nine missense mutations affecting exons 5–8, four mutations to STOP codon and three frameshift mutations due to single-base pair insertion or deletion in exons 3–5. Twenty out of the 24 cases have been previously reported in a study on *p53* and *p14ARF* abnormalities in OSCC (Bradley *et al*, 2001). There was no correlation between *Pidd* mRNA levels and *p53* mutation status. Figure 5 illustrates the distribution of *Pidd* expression in OSCC with and without *p53* mutation, and in normal oral epithelium from patients with no history of OSCC. Tumours with *p53* mutation showed a wide range in *Pidd* expression, with greater than 10-fold difference between the lowest and highest expression (Table 2).

## DISCUSSION

Our finding that *Pidd* expression in OSCC was associated with apoptosis represents the first demonstration of a link between *Pidd* and apoptosis in clinical tumour samples. Pidd has been characterised in mouse and human cell lines as a critical component of apoptotic signalling in response to DNA damage (Lin *et al*, 2000; Berube *et al*, 2005). *In vitro* studies demonstrated that exogenous expression of Pidd results in apoptosis and that Pidd interacts with two other apoptotic signalling proteins, FADD (Fas-associated death domain protein) and RAIDD (receptor-interacting protein-associated ICH-1/CED-3 homologous protein with a death domain). The results of these studies suggest that Pidd mediates apoptosis through activation of the FADD–caspase 8 and the RAIDD–caspase 2 complexes, and thus contributes to both the mitochondrial and death receptor signalling pathways (Tinel and Tschopp, 2004; Berube *et al*, 2005). The data shown here suggest that Pidd may be one of the factors that mediate apoptosis in OSCC, but further studies are needed to clarify the apoptotic signalling pathways that are activated in this context.

Tumour growth is the result of disruption of the normal balance between cell proliferation and cell death. The mechanisms through which this balance is disrupted determine the characteristics and evolution of the tumour (Sanchez-Beato *et al*, 2003). Our study of Pidd expression and apoptosis has revealed a subset of OSCC with a well-differentiated histological appearance that is associated with low Pidd expression, low AI and low PI. These tumours may be analogous to the ‘low-growth fraction B-cell lymphomas’ that include follicular lymphoma and B-cell chronic lymphocytic leukaemia, which are distinguished by resistance to apoptosis. In these malignancies, tumour growth is predominantly the result of abnormal cell survival since the PI is low (Sanchez-Beato *et al*, 2003). Tumour cells can acquire resistance to apoptosis by various mechanisms that interfere at different levels of apoptosis signalling and involve both the death receptor and mitochondrial pathways (Igney and Krammer, 2002). Previous studies of apoptosis in OSCC have described a correlation between high AI and well-differentiated carcinomas (Xie *et al*, 1999) and between the antiapoptotic protein Bcl-2 and high histological grade (less differentiated carcinomas) (Jordan *et al*, 1996; Stoll *et al*, 2000). Our data indicate the opposite relationship of low AI in well-differentiated tumours, and provide an explanation for tumour cell accumulation in well-differentiated carcinomas that have a low proliferation rate. Further studies of the apoptotic pathway that is mediated by Pidd should increase our understanding of the apoptotic defect that we observed in well-differentiated OSCC. Restoration of this apoptotic pathway may provide a selective approach to eradicate this subset of OSCC (Reed, 2006).

Our study has revealed another subset of OSCC that is characterised by a non-keratinising histological appearance, high Pidd expression, high AI and high PI. Studies of tumour cell lines and mouse tumour models have shown that oncogene activation and aberrant proliferation can trigger apoptosis through coupling of proliferative signalling pathways with apoptotic pathways (Igney and Krammer, 2002; Lowe *et al*, 2004). Abnormal proliferation may also trigger apoptosis in tumour cells because of hypoxia, nutrient deprivation, detachment from the extracellular matrix and adjacent cells, telomere shortening or other forms of DNA damage (Jaattela, 2004). Inactivation of apoptosis is thought to be required for tumour initiation and progression (Igney and Krammer, 2002; Jaattela, 2004; Lowe *et al*, 2004). However, studies of clinical tumour samples indicate that apoptosis may not be completely inactivated even in established malignancies. High-grade lymphomas such as lymphoblastic lymphoma and Burkitt's lymphoma are characterised by high PI and high AI. A positive correlation between PI and AI has been demonstrated for some types of malignant tumours (Kiberu *et al*, 1996; Zhang and Takenaka, 2000; Bai *et al*, 2003). These findings suggest that rapidly proliferating tumour cells may tolerate continuing apoptosis, when there is a high proliferation rate to allow net tumour expansion despite cell loss from apoptosis. Our data indicate that increased expression of *Pidd* may serve as a marker of activated apoptosis in a subset of OSCC.

In this study, *Pidd* expression was measured by quantitative RT-PCR in microdissected samples. This provided a sensitive and specific measurement of *Pidd* expression, but did not allow *Pidd* expression to be studied at the single-cell level. We performed immunohistochemical staining of selected cases of OSCC using an antiserum against Pidd (Berube *et al*, 2005) but were not able to demonstrate specific staining for Pidd. *In situ* hybridisation of tumour sections for *Pidd* mRNA was also unsuccessful, probably due to the low level of expression within individual cells.

We did not observe a significant difference in disease-free survival of patients with low or high *Pidd* expression in their carcinoma. Likewise, no difference in disease-free survival could be demonstrated for patients with OSCC of low or high AI. These findings suggest that *Pidd* expression and AI by TUNEL assay cannot predict the outcome of treatment in patients with OSCC who have been treated with surgical resection as the primary approach. Apoptotic cell death may be a more significant factor in determining tumour cell death and success of cancer treatment when radiation and/or cytotoxic drugs are used as primary therapy (Homma *et al*, 1999). Further study of patients with OSCC who are treated with primarily with radiation and/or chemotherapy would be helpful to determine whether *Pidd* expression and AI can serve as biological markers to indicate responsiveness to treatment.

*Pidd* was first described as a p53 target gene whose expression may be directly induced by p53. An association between *Pidd* expression and *p53* status was demonstrated in different cell types (Lin *et al*, 2000). Surprisingly, our data did not reveal a correlation between *Pidd* mRNA levels and *p53* mutation status. In particular, there were tumours with *p53* mutation that showed high *Pidd* expression. The lack of correlation between *p53* status and *Pidd* expression in OSCC suggests that *Pidd* is regulated by factors other than p53 in this tumour type. The expression of two other p53 target genes, *p21* and *Bax*, have been examined in head and neck cancer samples in relation to their *p53* status. There was no correlation between *p53* status and the expression of *p21* or *Bax* (Lavieille *et al*, 1998; Hotz *et al*, 1999; Osman *et al*, 2002; Xie *et al*, 2002). These findings suggest that, whereas the p53 tumour suppressor can induce numerous genes involved in growth arrest and apoptosis, these genes are also regulated by p53-independent mechanisms in a network of signalling pathways. This argues against a linear model of p53 function in which the level of expression of a p53 target gene such as *p21*, *Bax* or *Pidd* can be used to indicate p53 transcriptional activity.

## CONCLUSION

The expression of *Pidd* correlates with apoptosis in OSCC. Analysis of *Pidd* expression led to identification of subsets of OSCC that are distinguished by differences in differentiation, AI and PI.

## Figures and Tables

**Table 1:** Pidd expression, apoptotic index and proliferative index in OSCC according to disease stage (UICC staging system, 2002)

	**Stage II OSCC (N=15)**		**Stage III OSCC (N=12)**		**Stage IV OSCC (N=16)**	
	**Median (±standard deviation)**	**25th–75th Quartiles**	**Median (±standard deviation)**	**25th–75th Quartiles**	**Median (±standard deviation)**	**25th–75th Quartiles**
*Pidd* expression	1.33 (±0.46)	1.01–1.62	1.58 (±1.08)	0.82–1.98	1.69 (±1.18)	0.91–1.92
Apoptotic index	0.38 (±0.24)	0.22–0.49	0.52 (±0.57)	0.20–0.76	0.43 (±0.34)	0.18–0.66
Proliferative index	25 (±8.70)	15–30	24 (±7.72)	18–30	31 (±12.43)	14–38

OSCC=oral squamous cell carcinoma.

There are no significant differences among OSCC of different disease stages in Pidd expression, apoptotic index or proliferative index (Kruskal–Wallis test for Pidd expression, apoptotic index and proliferative index: *P*=0.465, *P*=0.404 and *P*=0.459, respectively).

**Table 2:** p53 mutation status and Pidd expression in oral squamous cell carcinomas

**Sample**	**p53 mutation status^a^**	**Pidd expression^b^**
1	Exon 5 Arg175His^c^	0.36
2	Exon 5 Val172Asp	0.49
3	Exon 4 Trp91Stop	0.58
4	Exon 5 Tyr163His	0.60
5	Exon 3 32ins1bp	0.64
6	Exon 6 Glu204Stop	0.67
7	Exon 6 Glu204Stop	0.68
8	Exon 8 Glu285Val	0.72
9	Exon 8 Pro278Leu	0.96
10	Exon 7 Tyr236Stop	0.88
11	Exon 5 Ser127Phe	0.92
12	Exon 6 Tyr220Cys	1.10
13	Exon 6 Tyr220Cys	1.22
14	Exon 4 88del1bp	1.71
15	Exon 8 Arg280Ser	1.92
16	Exon 5 161del1bp	4.69
17	No mutation detected	0.60
18	No mutation detected	0.79
19	No mutation detected	0.81
20	No mutation detected	0.83
21	No mutation detected	0.97
22	No mutation detected	0.97
23	No mutation detected	1.49
24	No mutation detected	2.59

a Determined by direct sequencing of the p53 gene from tumour samples.

b Pidd mRNA levels were measured by real-time RT-PCR and normalised to the expression of 18S RNA. The normalised expression is given in relative units, where the expression in the oral squamous carcinoma cell line UT-SCC24A is set at 1.

c The number refers to the affected codon.
